# Stimulation of Sigma-1 Receptor Protects against Cardiac Fibrosis by Alleviating IRE1 Pathway and Autophagy Impairment

**DOI:** 10.1155/2021/8836818

**Published:** 2021-01-04

**Authors:** Jing Qu, Miaoling Li, Dongxu Li, Yanguo Xin, Junli Li, Song Lei, Wenchao Wu, Xiaojing Liu

**Affiliations:** ^1^Laboratory of Cardiovascular Diseases, Regenerative Medicine Research Center, West China Hospital, Sichuan University, Chengdu 610041, China; ^2^Key Laboratory of Medical Electrophysiology of Ministry of Education, Institute of Cardiovascular Research, Southwest Medical University, Luzhou 646000, China; ^3^Department of Cardiovascular Surgery, West China Hospital, Sichuan University, Chengdu 610041, China; ^4^Department of Cardiology, West China Hospital, Sichuan University, Chengdu 610041, China; ^5^Department of Pathology, West China Hospital, Sichuan University, Chengdu 610041, China

## Abstract

Sigma-1 receptor (Sig1R), a chaperone in the endoplasmic reticulum (ER) membrane, has been implicated in cardiac hypertrophy; however, its role in cardiac fibroblast activation has not been established. This study investigated the possible association between Sig1R and this activation by subjecting mice to sham, transverse aortic constriction (TAC), and TAC plus fluvoxamine (an agonist of Sig1R) treatments. Cardiac function and fibrosis were evaluated four weeks later by echocardiography and histological staining. In an *in vitro* study, neonatal rat cardiac fibroblasts were treated with fluvoxamine or NE-100 (an antagonist of Sig1R) in the presence or absence of transforming growth factor beta1 (TGF-*β*1). Fibrotic markers, ER stress pathways, and autophagy were then investigated by qPCR, western blotting, immunofluorescence, confocal microscopy, and transmission electron microscopy. Fluvoxamine treatment reduced cardiac fibrosis, preserved cardiac function, and attenuated cardiac fibroblast activation. Inhibition of the IRE1/XBP1 pathway, a branch of ER stress, by a specific inhibitor of IRE1 endonuclease activity, attenuated the pathological process. Fluvoxamine stimulation of Sig1R restored autophagic flux in cardiac fibroblasts, indicating that Sig1R appears to play a protective role in the activation of cardiac fibroblasts by inhibiting the IRE1 pathway and restoring autophagic flux. Sig1R may therefore represent a therapeutic target for cardiac fibrosis.

## 1. Introduction

Cardiac fibrosis is characterized by the cardiac fibroblast activation, excessive proliferation, and transition into myofibroblast, which lead to excessive deposition and abnormal distribution of extracellular matrix [1–3]. Cardiac fibrosis usually happens postmyocardial infarction and myocardial hypertrophy, causing chronic heart failure finally [4, 5]. Cardiac fibrosis is a common pathological process in the development of various cardiovascular diseases and a risk for sudden cardiac death [6]. It is known that various cellular signaling pathways, such as the renin-angiotensin system, inflammatory factors, and oxidative stress are involved in the process of cardiac fibrosis, whereas the underling mechanisms, especially myocardial fibroblast activation, is not fully understood [7, 8]. Therefore, further exploring the pathophysiologic mechanism of cardiac fibrosis may provide new insights and be helpful for clinical treatment.

ER stress has gained attention as a cellular mechanism for maintaining homeostasis. It is elicited by the disruption of ER homeostasis and the accumulation of unfolded or misfolded proteins, followed by the activation of three sensors that subsequently activate downstream signaling pathways: Protein Kinase R-like ER kinase (PERK), Inositol Requiring Enzyme 1*α* (IRE1*α*), and Activating Transcription Factor 6 (ATF6) [9, 10]. Previous studies have confirmed the involvement of ER stress in the pathogenesis of cardiac hypertrophy [11, 12].

Other evidence has also shown that autophagy is critical for the development of cardiovascular diseases, such as cardiac hypertrophy and heart failure [13, 14]. Lysosome-mediated autophagy degrades and recycles cellular wastes, including proteins, lipids, and dysfunctional organelles. ATG-mediated autophagosomes/autolysosomes formation and autophagosome content degradation are key processes involved in autophagy [15].

A variety of autophagy proteins are localized at the endoplasmic reticulum (ER) [16], and autophagy originates from mitochondrial-associated endoplasmic reticulum membrane (MAM), the interface between ER and mitochondria [17, 18]. Sigma-1 receptor (Sig1R), a 223-amino acid ER chaperone at MAM, is related to autophagy and ER stress [19–21].

Sig1R modulates ER stress, autophagy, and apoptosis and has been confirmed to participate in neurodegenerative diseases and cardiac hypertrophy [22–25]. Fluvoxamine, a selective serotonin reuptake inhibitor with high affinity for the Sig1R, ameliorates cardiac hypertrophy and dysfunction deriving from Sig1R activation [26–29]. While this finding introduces the role of Sig1R in modulating cardiovascular disease, it raises many questions regarding the underline mechanisms, especially in cardiac fibrosis.

Therefore, in this study, we determined how Sig1R regulates cardiac fibrosis and cardiac fibroblasts activation, as well as its roles in ER stress, autophagy.

## 2. Methods and Materials

### 2.1. TAC Surgery

In this study, all mice received humane care, and our study was approved by the animal ethics committee of West China Hospital of Sichuan University. TAC was performed to induce pressure overload in the mice's heart [30]. Briefly, male mice (6-8 weeks old, 20-25 g body weight, Beijing Vital River Laboratory Animal Co. Ltd. China) were anesthetized with 2% isoflurane inhalation. The animals were then placed in a supine position, intubated orally with a 20-gauge tube, and ventilated (Harvard Apparatus Rodent Ventilator, MiniVent) at 120 breaths per minute (0.1 ml tidal volume). A stitched 27-gauge needle was sutured on the aortic arch between the cephalic artery and left carotid artery to form a reproducible aortic valve stenosis. Control mice were sham-operated. In this study, at the beginning of the experiment, we used 25 C57BL/6 mice for TAC surgery and 6 mice for sham surgery. Five died during the operation of TAC surgery, and a total of 20 TAC mice survived after the operation. We randomly divided the mice after TAC surgery into the TAC group and the fluvoxamine intraperitoneal injection group (FLV group), 10 mice per group. Some mice have died during the feeding process after the end of TAC operation and during the intraperitoneal injection administration, so the statistics number of mice finally included in this study was *n* = 6 in each group.

### 2.2. Echocardiography

At 4 weeks after the TAC surgery, mice were lightly anesthetized with 1% isoflurane inhalation and subjected to echocardiography using a Vevo3100 instrument (Visual Sonics). Images were captured in the short axis of the left ventricle to calculate internal wall dimensions during systole and diastole. From M-mode images, the thickness and dimensions of the left ventricle (LV) chamber were obtained. LV systolic function was determined by calculating ejection fraction (EF) and fractional shortening (FS). Echocardiography was performed on all mice.

### 2.3. Histological Staining

Heart tissue was rinsed with ice-cold saline perfusion and then with 0.1 ml of 10% KCl to cause diastolic arrest. The heart tissue was then fixed in 4% paraformaldehyde for 1 week at 4°C, then paraffin-embedded and sectioned at 5 *μ*m. The heart sections were dewaxed and hydrated through a graded ethanol series (100, 95, 75, and 50%) and then stained either with Sirius red and Masson trichrome to observe fibrosis or hematoxylin and eosin (HE) to show the heart structure.

### 2.4. Quantitative Real-Time PCR (qRT-PCR)

The classic TRIzol (Invitrogen, USA) method was performed to extract total RNA from tissues or cells. Next, the RNA was used as a template to synthesize cDNA with a reverse transcription. The reaction system (ReverTra Ace qPCR RT Kit, FSQ-101, TOYOBO) is displayed in Table 1. The q-PCR was conducted using the SYBR Green Supermix kit (Bio-Rad, USA) on BIO-RADCFX96™ Real-Time PCR Detection System and *β*-actin served as the reference gene. The primers used are displayed in Tables 2 and 3. Relative fold expression values were determined by applying the ^△△^CT threshold (Ct) method.

### 2.5. Protein Isolation and Western Blotting

Protein lysates were collected from mice heart tissue or cardiac fibroblasts and prepared for western blots as previously reported [31]. Briefly, 20-30 *μ*g protein was separated on 10% or 15% SDS-PAGE gels and transferred to PVDF membranes. After blocking by 5% skim milks, blots were incubated with primary antibodies. After incubation with corresponding anti-mouse/rabbit secondary antibodies (1 : 3000; ZSGB-BIO), immunoblots were developed using Chemiscope 6000 (CLINX, China). The relative protein expressions were analyzed by ImageJ software. GAPDH or *β*-Actin was served as an internal reference.

### 2.6. Cell Culture and Pharmaceutical Treatments

Neonatal rat cardiac fibroblasts were isolated from the hearts of decapitated Sprague-Dawley rats according to the methods described previously [32]. The CFs were grown in a culture flask with DMEM mixed with 10% FBS and 100 U/ml of both streptomycin and penicillin. CFs at the second passaging were added into 6-well plates and cultivated to reach 50% confluence. The CFs were incubated for 4-6 hours with serum-free DMEM and then later treated with TGF-*β*1 (a known stimulator of CF, 10 ng/ml, Sino Biological Inc.) for 24 h to induce fibroblasts activation. A subset of cells was treated with fluvoxamine (5 *μ*M) or NE-100 (5 *μ*M) before 2 h exposure to TGF-*β*1.

### 2.7. Immunofluorescence Staining and Confocal Microscopy

When the confluence of cardiac fibroblasts reached 90%, the cells were digested with trypsin, centrifuged, and counted. After inoculating cardiac fibroblasts into a 24-well glass culture plate and cultivating it to a density of 50%, according to the purpose of the experiment, the corresponding drugs such as TGF-*β*1 and fluvoxamine were stimulated for 24 h, then the medium was discarded, and precooled PBS rinse 3 times; 4% paraformaldehyde-fixed for 30 min; 0.5% Triton X-100 permeates the cells. Incubate primary antibody *α*-SMA (1 : 200) or Sig1R (1 : 200) overnight; the next day, incubate secondary antibody: secondary antibody (anti-rabbit Alexa Fluor 488-conjugated secondary antibody, Invitrogen, USA), diluted 1 : 1000 in PBS, incubated at room temperature in the dark for 2 hours. Nuclei staining: cells were incubated with DAPI (1 : 1000), diluted in PBS, and incubated at room temperature for 5 min. Images were collected using a Laser Scanning Confocal Microscopy (FluoView™ FV1000, OLYMPUS, Japan) and analyzed with ImageJ software.

### 2.8. EdU Assay to Detect Cell Proliferation

Proliferation was detected using EdU Assay Kit (RibobilTM, China) in the cardiac fibroblast as described [33]. The proportion of EdU-incorporated cells was defined as the proliferation rate. The proliferation rate was calculated by normalizing the number of EdU positive cells to the DAPI-stained cells under the fluorescence microscope (Nikon, Japan). Each assay was performed at least three times. Cell proliferation rate = number of EdU − incorporated cells/total number of cells.

### 2.9. Wound Healing Assay

Wound-healing assays were used to measure the migration of CFs according to our previous report [30]. When the cell confluence in the 6-well plate reaches 50%, treat it before scratching: use a 200 *μ*l sterile tip to scratch and take a picture at the bottom of the well plate, and take another picture at the same position after the stimulation for comparison. 6 fields of vision were collected in each group, and ImageJ software was used to determine the scratch area. Cell migration rate = (0 h scratch area − 24 h scratch area)/0 h scratch area.

### 2.10. Small Interfering RNA (siRNA) Transfection

Cells grown to 40-50% confluence were transferred to 6-well plates. They were transfected using transfection reagent riboFECT™ CP (RiboBio™, China). siRNA targeting Sig1R (si-Sig1R) was transfected into NRCFs for 24 hours then treated with TGF-*β*1 for 48 hours. Individual siRNAs (100 nM, RiboBio™, China), ribo-FECT™ CP reagent and buffer, and DMEM were combined and then incubated for 15 minutes at room temperature. The experiment was divided into four groups: siNC group, siNC+T group, si-Sig1R group, and si-Sig1R+T group.

### 2.11. mRFP-GFP-LC3 Adenovirus Transfection

Autophagy was detected by mRFP-GFP-LC3 adenovirus transfection [34]. Cells were transfected with mRFP-GFP-LC3 adenovirus (Hanbio Biotech, Shanghai, China) for 24 h and then pretreated with fluvoxamine or NE-100, prior to TGF-*β*1 administration. Treated cells were fixed with 4% paraformaldehyde in PBS, and images were obtained using a laser scanning confocal microscope. Merged fluorescence from RFP and GFP was analyzed with Pearson's correlation coefficient, and 15 cells were used for quantification in each group.

### 2.12. Transmission Electron Microscopy Assay

TEM assay was performed as our previous study [30]. Cardiac fibroblasts were washed in precold PBS and then fixed in cold 2.5% glutaraldehyde for 2 h at 4°C; cells were washed with PBS (0.2 mol/L, pH 7.4) for 2 h, fixed with 1% osmic acid for 2 h, and then washed six times with PBS for 10 min per wash. The samples were dehydrated with ethanol and cleaned with epoxypropane. They were embedded in EPON812 overnight at room temperature. Ultrathin sections (40–60 nm) were cut (EM UC61rt, Leica) and stained with uranyl acetate/lead citrate. Autophagosomes and autolysosomes were observed using a transmission electron microscope from Hitachi (H-7650).

### 2.13. Antibodies and Reagents

Antibodies used in this study included anti-Sig1R (Abcam, ab53852), anti-POSTN (Abcam, ab14041), anti-*α*-SMA (Abcam, ab32575), anti-CTGF (Abcam, ab6992), anti-TGF-*β* (Abcam, ab92486), anti-ATF4 (Cell Signaling Technology, 11815), anti-p-PERK (Cell Signaling Technology, 3173), anti-IRE1*α* (Cell Signaling Technology, 3294), anti-Xbp1s (Cell Signaling Technology, 82914), anti-LC3B (Abcam, ab92486), anti-ATG7 (Cell Signaling Technology, 8558), anti-GAPDH (Cell Signaling Technology, 5174), anti-P62 (Cell Signaling Technology, 88588), and anti-c-ATF6 (Abcam, ab62576), all obtained from rabbits, and anti-*β*-Actin (Cell Signaling Technology, 3700) from mice. The following reagents were used: TGF- *β*1 (10 ng/ml, Sino Biology), fluvoxamine (MCE, HY-B0103A), NE-100 (Sigma-Aldrich, SML0631), 4*μ*8C (MCE, HY-19707), thapsigargin (MCE, HY-13433), and 4-PBA (Sigma-Aldrich, SML0309).

### 2.14. Statistics

The data was shown as the average of at least 3 independent experiments (mean ± SEM). Student's *t*-test was used to compare two data sets and analyze the variance of multiple data sets. Significance is defined as *p* < 0.05. The statistical software used is Prism v.7.

## 3. Results

### 3.1. The Expression of Sig1R Is Decreased in Fibrotic Heart Tissues of TAC Mice and in Activated Cardiac Fibroblasts

**Figure 1:**
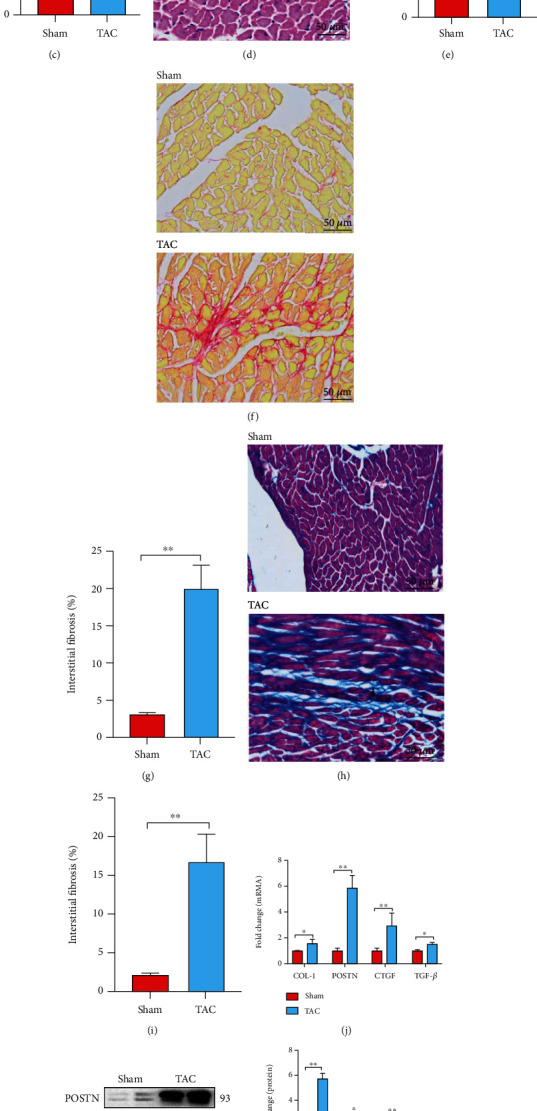
Sig1R is downregulated in mice heart tissue following transverse aortic constriction (TAC) surgery. Mice were randomly divided into two groups: Sham operation and TAC. (a, b) Cardiac function decline and cardiac hypertrophy were evaluated by echocardiography shown by percent EF (ejection fraction) and FS (fractional shortening); diastole IVS (Interventricular Septal) and LVPW (left ventricular posterior wall) thickness. *n* = 6; (c) Cardiac hypertrophy index, HW/BW (heart weight to body weight ratio). *n* = 6; (d, e) Representative cross-sectional images of hematoxylin/eosin-stained cardiomyocytes. Scale bar = 50 *μ*m. *n* = 6. (f–i) Heart sections were stained with Sirius red and Masson trichrome to visualize fibrosis (red and blue). Scale bar = 50 *μ*m. *n* = 6. (j) The mRNA levels of COL-1(collagen I), POSTN (periostin), *α*-SMA (*α*-Smooth Muscle Actin), CTGF (connective tissue growth factor), and TGF-*β* (transforming growth factor-*β*) in mice heart tissue. *n* = 6; (k, l) The protein levels of POSTN, *α*-SMA (*α*-Smooth Muscle Actin), CTGF, and TGF-*β* in mice heart tissue. *n* = 6; (m–o) The mRNA and protein levels of Sig1R (Sigma-1 receptor) in mice heart tissue. *n* = 6. Shown are representative pictures; statistical significance was determined by unpaired *t*-test. ^∗^*p* < 0.05, ^∗∗^*p* < 0.01, ^∗∗∗^*p* < 0.001. Data represent the mean ± SEM.

To understand the expression of Sig 1R in pathological myocardium, we established a cardiac hypertrophy model with TAC surgery. The TAC mice versus sham-operated mice revealed obvious cardiac function decline (Figures 1(a) and 1(b)) and a higher heart weight to body weight ratio (Figure 1(c)). Cardiac function of TAC mice was significantly decreased as shown by lower fractional shortening (FS), ejection fraction (EF), diastolic interventricular septum (IVS), and left ventricular posterior wall (LVPW) thickness (Figure 1(b)). Hematoxylin/Eosin (HE) staining showed the TAC mice model exhibited significant cardiac hypertrophy (Figures 1(d) and 1(e)). Sirius red (Figures 1(f) and 1(g)) and Masson trichrome (Figures 1(h) and 1(i)) staining showed that cardiac fibrosis was successfully induced in our TAC model. The mRNA expression of cardiac fibrosis markers, collagen I (COL-1), periostin (POSTN), connective tissue growth factor (CTGF), and transforming growth factor-*β* (TGF-*β*) was significantly increased by 1.5-, 5.8-, 2.9-, and 1.5-fold, respectively, when compared with expression in the Sham group (Figure 1(j)). The protein expressions of POSTN, *α*-SMA, CTGF, and TGF-*β* were increased by 5.6-, 2.5-, 2.4-, and 2.2-fold, respectively, in the TAC group compared with the Sham group (Figures 1(k) and 1(l)). Simultaneously, the mRNA and protein expressions of Sig1R were decreased by 30% and 55%, respectively, compared to expressions in the Sham group (Figures 1(m) and 1(o)).

**Figure 2:**
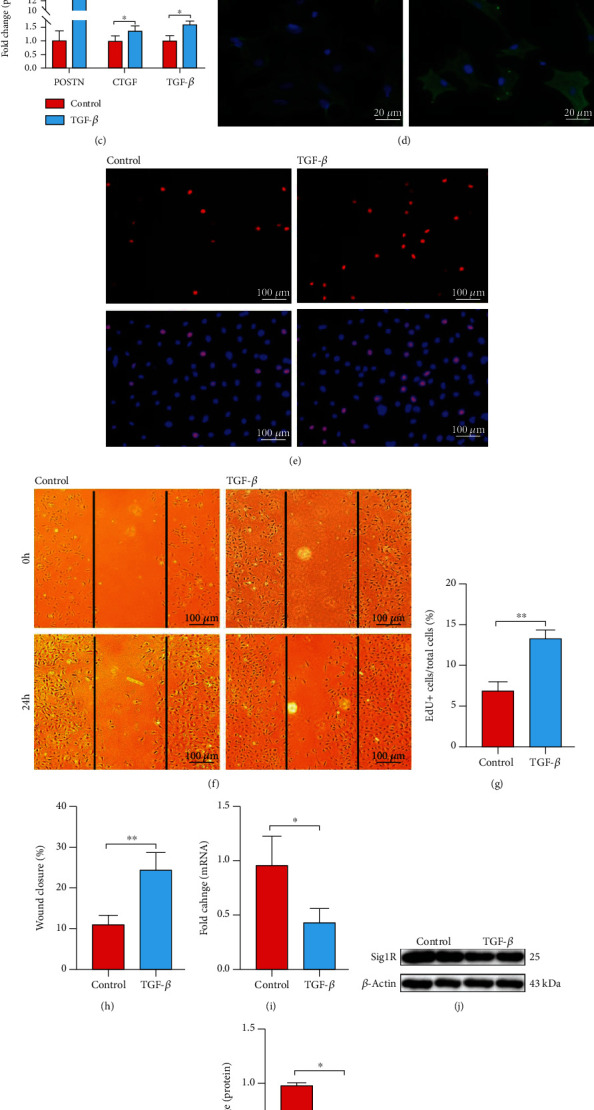
Sig1R is downregulated in the activation of cardiac fibroblasts induced by TGF-*β*1. Cardiac fibroblasts were randomly divided into two groups. (a) The q-PCR results of COL-1, POSTN, CTGF, and TGF-*β* in cardiac fibroblasts from control and TGF-*β*1 treatment (TGF-*β*) groups. *n* = 4; (b, c) The representative western blot results of POSTN, CTGF, and TGF-*β* in cardiac fibroblasts from control and TGF-*β*1 treatment (TGF-*β*) groups. *n* = 3; (d) Representative images of *α*-SMA fluorescence of cardiac fibroblasts were shown (the green fluorescence indicates *α*-SMA and the blue fluorescence indicates the nucleus stained by DAPI). Scale bar = 20 *μ*m, *n* = 100; (e, g) The proliferation rate of cardiac fibroblasts was assessed by EdU assay (the red fluorescence indicates cells that incorporated EdU and the blue fluorescence indicates the nucleus stained by Hoechst 33342). Scale bar = 100 *μ*m, *n* = 200; (f, h) Scratch wound-healing assay showing cardiac fibroblast migration; images were taken at 0 and 24 h postscratch. Black lines denote the wound borders. Scale bar = 100 *μ*m. *n* = 6; (i–k) The mRNA levels of Sig1R were assessed by q-PCR. *n* = 4; (j, k) The representative western blot result of Sig1R in cardiac fibroblasts from Control and TGF-*β*1 treatment (TGF-*β*) groups. *n* = 3; (l) Representative images of Sig1R fluorescence of cardiac fibroblasts were shown (the green fluorescence indicates Sig1R expression and the blue fluorescence indicates the nucleus stained by DAPI). Scale bar = 5 *μ*m. *n* = 50. Shown are representative pictures, *p* was determined by unpaired *t*-test. ^∗^*p* < 0.05, ^∗∗^*p* < 0.01, ^∗∗∗^*p* < 0.001. Data represent the mean ± SEM.

The fibrotic markers in the activation of cardiac fibroblasts induced by fibrotic agonist TGF-*β*1 for 24 h are shown in Figure 2. Compared with the control group, the TGF-*β* group mRNA expressions of POSTN, COL-1, CTGF, and TGF-*β* were increased by 10.0-, 1.5-, 1.6-, and 1.5-fold (Figure 2(a)), respectively, and the TGF-*β* group protein expressions of POSTN, CTGF, and TGF-*β* increased by 15.0-, 1.4-, and 1.6-fold (Figures 2(b) and 2(c)), respectively. Additionally, immunofluorescence staining showed the upregulation of *α*-SMA induced by TGF-*β*1 in cardiac fibroblasts (Figure 2(d)). The proliferation (Figures 2(e) and 2(g)) and migration (Figures 2(f) and 2(h)) capacities were also increased in the TGF-*β*1-stimulated cardiac fibroblasts. Under the stimulated condition, the mRNA and protein expressions of Sig1R in the activation of cardiac fibroblasts were decreased by 58% and 30%, respectively, compared with the control group (Figures 2(i)–2(k)). Moreover, immunofluorescence staining confirmed the decrease of Sig1R in the activation of cardiac fibroblasts (Figure 2(l)). These results indicated that the expression of Sig1R was decreased during the process of cardiac fibrosis.

### 3.2. Stimulation of Sig1R Attenuates the Activation of Cardiac Fibroblasts In Vitro

**Figure 3:**
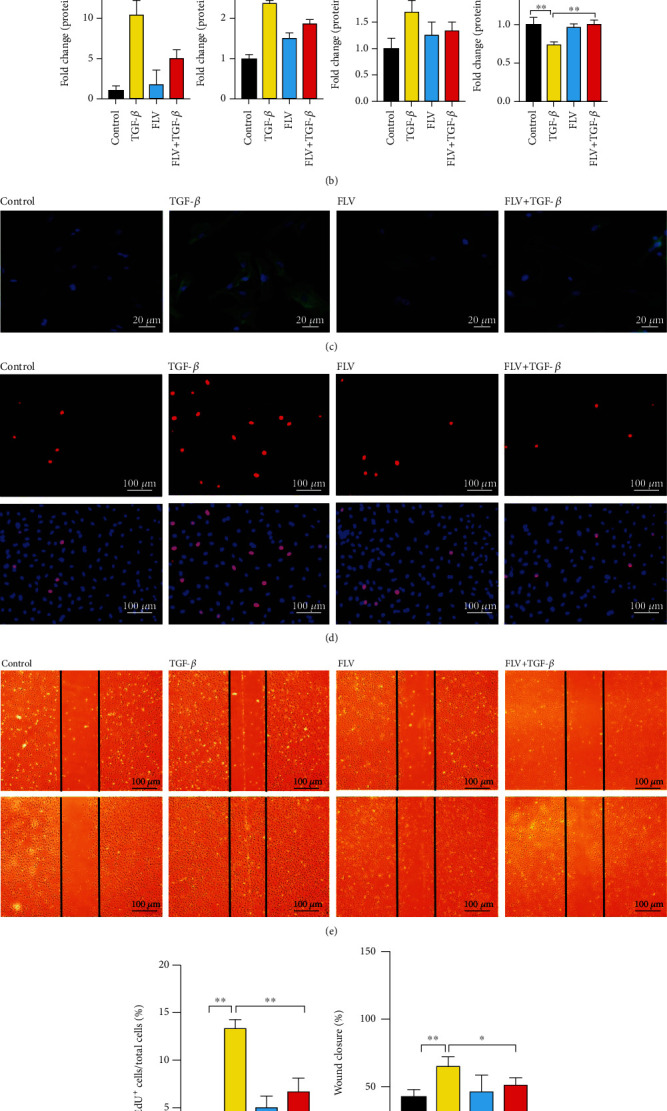
Stimulation of Sig1R attenuates cardiac fibroblast activation. Cardiac fibroblasts were randomly divided into four groups: control, TGF-*β*, FLV, and FLV+TGF-*β*. (a, b) The representative western blot results of POSTN, CTGF, and TGF-*β* in cardiac fibroblasts from control, TGF-*β*1 treatment (TGF-*β*) groups, fluvoxamine treatment (FLV), or fluvoxamine combined with TGF-*β*1 treatment (FLV+TGF-*β*) groups. *n* = 3; (c) Representative images of *α*-SMA fluorescence in cardiac fibroblasts from different groups. Scale bar = 20 *μ*m. *n* = 100; (d, f) The proliferation rate of cardiac fibroblasts from different groups was assessed by EdU assay. Scale bar = 100 *μ*m, *n* = 200; (e, g) Scratch wound-healing assay in cardiac fibroblasts from different groups; images were taken at 0 and 24 h postscratch. Black lines denote the wound borders. Scale bar = 100 *μ*m. *n* = 6; Shown are representative pictures, *p* was assessed by one-way ANOVA analysis. ^∗^*p* < 0.05, ^∗∗^*p* < 0.01, ^∗∗∗^*p* < 0.001. Data represent the mean ± SEM.

Pretreatment of cardiac fibroblasts with fluvoxamine for 2 h before TGF-*β*1-stimuli decreased the expressions of the fibrosis marker POSTN, CTGF, and TGF-*β* by 50%, 23%, and 22%, respectively (Figures 3(a) and 3(b)). Furthermore, immunofluorescence staining confirmed a significant reduction in *α*-SMA expression (Figure 3(c)). Evaluation of cardiac fibroblast proliferation by the EdU incorporation assay revealed a significant reduction in cell proliferation by fluvoxamine pretreatment in activated cardiac fibroblasts (Figures 3(d) and 3(f)). In addition, we found that fluvoxamine did not affect cell proliferation in cardiac fibroblasts, which is not treated with TGF-*β*1.

Fluvoxamine-pretreated cells also displayed a significant reduction in migration in the scratch-wound healing assay (Figures 3(e) and 3(g)). Taken together, these findings suggested that the stimulation of Sig1R has a potential role in diminishing myofibroblast proliferation and reducing cell migration, as well as ameliorating the activated myofibroblast phenotype.

**Figure 4:**
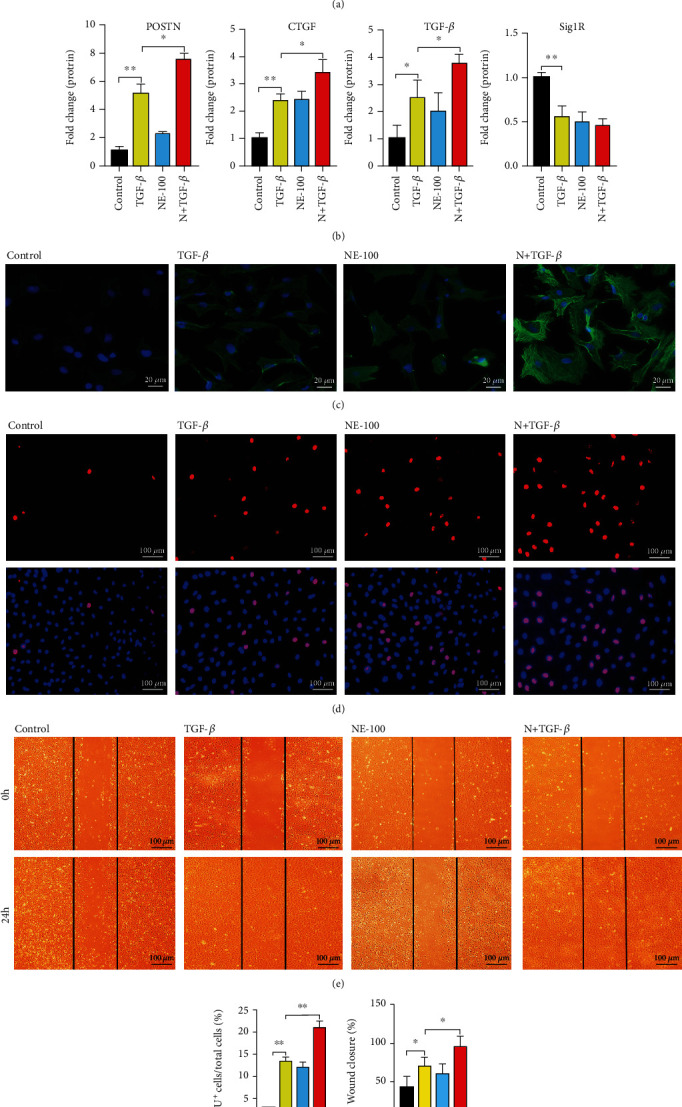
Inhibition of Sig1R further promotes cardiac fibroblast activation. Cardiac fibroblasts were randomly divided into four groups: control, TGF-*β*, NE-100, and NE-100+TGF-*β*1. (a, b) The representative western blot results of POSTN, CTGF, and TGF-*β* in cardiac fibroblasts from control, TGF-*β*1 treatment (TGF-*β*) groups, NE-100 treatment (NE-100), or NE-100 combined with TGF-*β*1 treatment (N+TGF-*β*) groups. *n* = 3; (c) Representative of immunofluorescence staining showed *α*-SMA (green) in cardiac fibroblasts from different groups. Scale bar = 20 *μ*m, *n* = 100 (d, f) The proliferation rate of cardiac fibroblasts from different groups was assessed by EdU assay. Scale bar = 100 *μ*m, *n* = 200; (e, g) Scratch wound-healing assay in cardiac fibroblasts from different groups; images were taken at 0 and 24 h postscratch. Black lines denote the wound borders. Scale bar = 100 *μ*m. *n* = 6. Shown are representative pictures, *p* was determined by one-way ANOVA analysis. ^∗^*p* < 0.05, ^∗∗^*p* < 0.01, ^∗∗∗^*p* < 0.001. Data represent the mean ± SEM.

The role for Sig1R in this pathological condition was further verified, as cardiac fibroblasts pretreated with NE-100, a Sig1R antagonist, prior to TGF-*β*1 administration showed a more active phenotype than cells treated only with TGF-*β*1. Western blot revealed the upregulation of protein expressions of POSTN, CTGF, and TGF-*β* by 1.5-, 1.6-, and 1.6-fold, respectively, in the N+TGF-*β* group (Figures 4(a) and 4(b)), and immunofluorescence staining also showed the increased protein expression of *α*-SMA in the N+TGF-*β* group compared with the control group (Figure 4(c)). NE-100 further promoted the proliferation (Figures 4(d) and 4(f)) and migration (Figures 4(e) and 4(g)) of activated cardiac fibroblasts, supporting a promotion of cardiac fibroblast activation by blocking of Sig1R activity.

**Figure 5:**
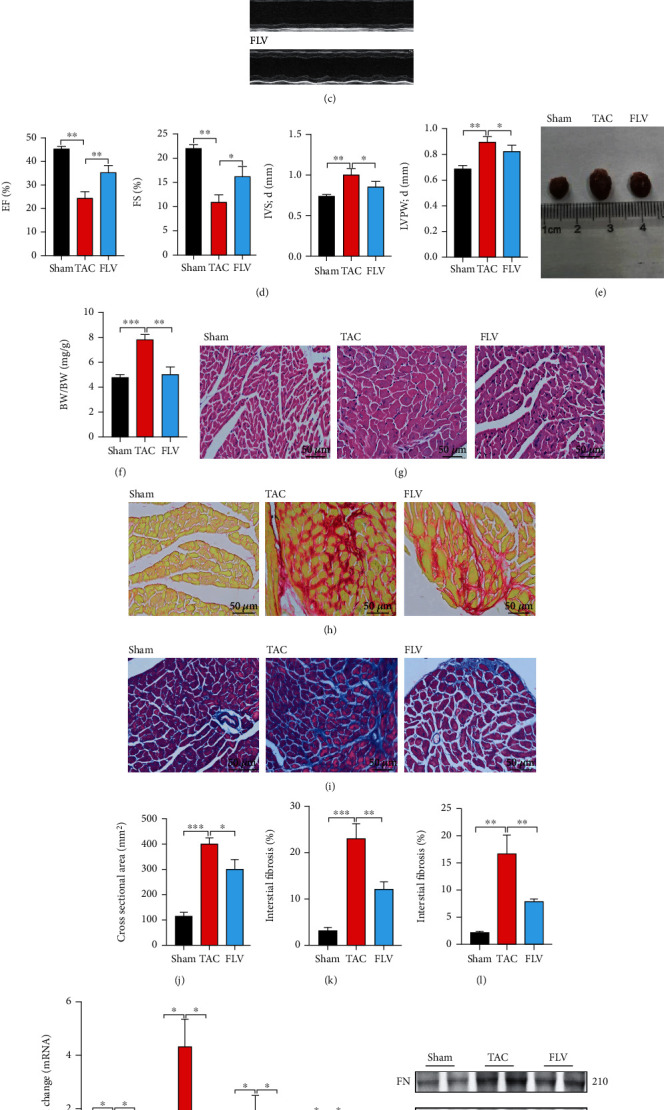
The siRNA of Sig1R further promotes cardiac fibroblast activation; treatment with the Sig1R agonist fluvoxamine reduces cardiac fibrosis and preserves cardiac function 4 weeks post-TAC. Mice were randomly divided into three groups: Sham, TAC, and FLV. (a, b) The protein levels of POSTN, CTGF, Sig1R, and TGF-*β* in activated cardiac fibroblasts transfected with Sig1R siRNA. *n* = 3; (c, d) Cardiac function and hypertrophy evaluated by echocardiography shown by percent EF and FS; diastole IVS and LVPW thickness. *n* = 6; (e, f) Cardiac images and cardiac hypertrophy index, HW/BW, *n* = 6; (g, j) Representative cross-sectional images of hematoxylin/eosin-stained cardiomyocytes. Scale bar = 50 *μ*m. *n* = 6. (h, i, k, and l) Representative images of Sirius red and Masson trichrome staining of heart tissue are shown to visualize fibrosis (red and blue). Scale bar = 50 *μ*m. *n* = 6; (m) The mRNA levels of COL-1, POSTN, CTGF, and TGF-*β* in mice heart tissue. *n* = 6; (n, o) The protein levels of FN (Fibronectin), POSTN, CTGF, and TGF-*β* in mice heart tissue. *n* = 6. Shown are representative pictures, *p* value was determined by one-way ANOVA analysis. ^∗^*p* < 0.05, ^∗∗^*p* < 0.01, ^∗∗∗^*p* < 0.001. Data represent the mean ± SEM.

To further determine the role of Sig1R in the activation of cardiac fibroblast and exclude the several off-target effects of small molecule inhibitors, we used Sig1R siRNA to specifically silence the expression of Sig1R under basic conditions or stimulated by TGF-*β*1. As shown in Figures 5(a) and 5(b), compared with the negative control group (siNC+T group) treated with TGF-*β*1, after silencing Sig1R and then TGF-*β*1 stimulating for 24 h (si-Sig1R + T group). Cardiac fibroblast activation protein marker POSTN, CTGF, and TGF-*β* were increased by 1.4-, 1.3-, and 1.3-fold, respectively. The results indicated that the silencing of Sig1R expression by siRNA further promoted the activation of cardiac fibroblast. This result is consistent with the effect of small molecule inhibitors.

Collectively, these data demonstrated that the stimulation of Sig1R might be a therapeutic candidate for cardiac fibroblast activation.

### 3.3. Treatment with Sig1R Agonist Reduces Mice Cardiac Fibrosis and Preserves Cardiac Function

Mice injected intraperitoneally with fluvoxamine (1 mg/kg) once daily [35] for 4 weeks consecutive days after TAC operation showed changes in cardiac function and dimensions detectable by echocardiography (Figures 5(c)–5(f)). Cardiac dysfunction was observed in TAC mice in the form of reduced FS and EF and increased IVS and LVPW (Figure 5(d)) when compared with the sham group. Interestingly, fluvoxamine-treated animals exhibited an attenuation of cardiac function decline in terms of pathologic hypertrophy (Figures 5(d)–5(g)). This observed attenuation of cardiac hypertrophy was confirmed by a reduction in the cell size in heart tissue after fluvoxamine administration (Figures 5(e)–5(g) and 5(j)). Fluvoxamine treatment also decreased collagen deposition, as shown by Sirius red (Figures 5(h) and 5(k)) and Masson (Figures 5(i) and 5(l)) staining. Q-PCR (Figure 5(m)), and western blotting (Figures 5(n) and 5(o)) confirmed a reduction in the levels of fibrotic markers in the LV at 4 weeks after TAC. Taken together, these data indicated that Sig1R treatment helped to preserve cardiac function and attenuated cardiac fibrosis after TAC.

### 3.4. Sig1R Regulates ER Stress through Inhibition of the IRE1*α* Signaling in Activated Cardiac Fibroblasts

**Figure 6:**
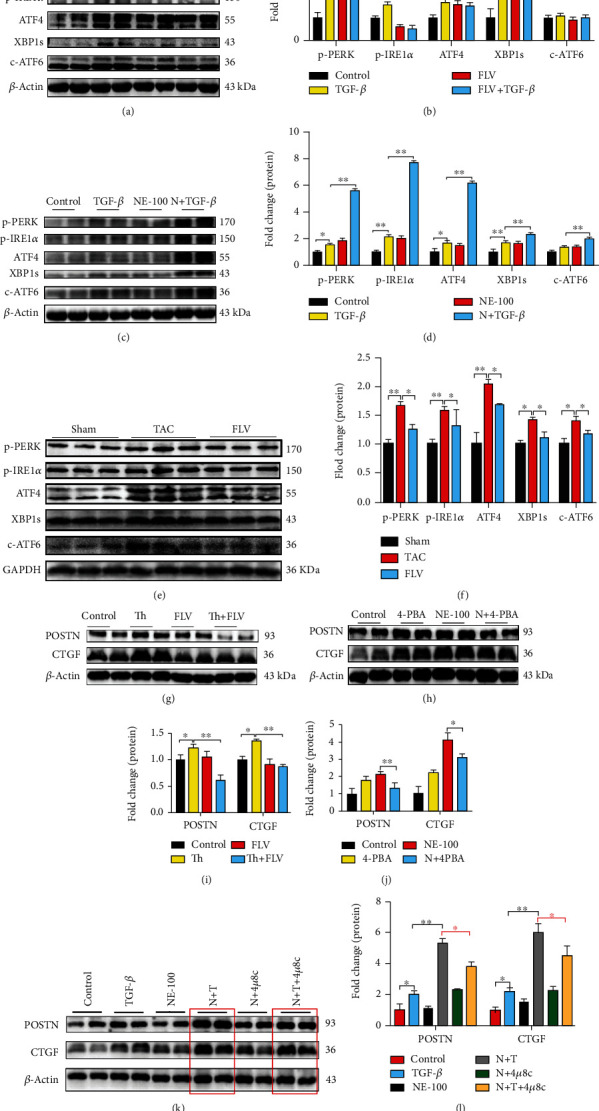
Sig1R protects against cardiac fibrosis by inhibition of ER stress. (a, b) The representative western blot results of p-PERK, p-IRE1*α*, ATF4, XBP1s, and c-ATF6 in cardiac fibroblasts from control, TGF-*β*1 treatment (TGF-*β*) groups, fluvoxamine treatment (FLV), or fluvoxamine combined with TGF-*β*1 treatment (FLV+TGF-*β*) groups. *n* = 3; (c, d) The representative western blot results of p-PERK, p-IRE1*α*, ATF4, XBP1s, and c-ATF6 in cardiac fibroblasts from control, TGF-*β*1 treatment (TGF-*β*) groups, NE-100 treatment (NE-100), or NE-100 combined with TGF-*β*1 treatment (N+TGF-*β*) groups. *n* = 3; (e, f) The representative western blot results of p-PERK, p-IRE1*α*, ATF4, XBP1s, and c-ATF6 in heart tissue from sham-operated (Sham), TAC, and intraperitoneal injection with fluvoxamine after TAC (FLV) groups. *n* = 6; (g, i) The representative western blot results of POSTN and CTGF in cardiac fibroblasts from control, thapsigargin treatment only (Th), fluvoxamine treatment, and thapsigargin combined fluvoxamine treatment (Th+FLV) groups. *n* = 3; (h, j) The representative western blot results of POSTN and CTGF in cardiac fibroblasts from control, 4-PBA treatment only (4-PBA), NE-100 treatment (NE-100), and NE-100 combined 4-PBA treatment (N+4-PBA) groups. (k, l) The protein levels of POSTN and CTGF in cardiac fibroblasts from different groups. *n* = 3. Shown are representative pictures, *p* value was determined by one-way ANOVA with Tukey post hoc analysis. ^∗^*p* < 0.05, ^∗∗^*p* < 0.01, ^∗∗∗^*p* < 0.001. Data represent the mean ± SEM.

The decreased expression of Sig1R in TGF-*β*1-activated cardiac fibroblasts (Figures 6(a)–6(d)) and fibrotic hearts (Figures 6(e) and 6(f)) was associated with ER stress in the present study. As shown in Figures 6(a)–6(d), the administration of TGF-*β*1 upregulated the levels of the ER stress markers phosphorylated IRE1*α* (p-IRE1*α*), spliced Xbp1 (Xbp1s), phosphorylated PERK (p-PERK), ATF4, and cleaved ATF6 (c-ATF6). By contrast, pretreatment with fluvoxamine reversed these inductions (Figures 6(a) and 6(b)), whereas NE-100 pretreatment exacerbated the inductions (Figures 6(c) and 6(d)). ER stress was also activated in the mice model of pressure overload-induced cardiac fibrosis, as shown in Figures 6(d) and 6(f). After TAC surgery, fluvoxamine was injected intraperitoneally for 4 consecutive weeks (FLV group). The protein expression levels of p-PERK, p-IRE1*α*, ATF4, XBP1s, and c-ATF6 were reduced by 30%, 22%, 25%, 18%, and 15%, respectively (Figures 6(e) and 6(f)). Treatment with fluvoxamine followed by treatment with the ER stress activator thapsigargin decreased the protein expression levels of POSTN and CTGF when compared with thapsigargin treatment only (Figures 6(g) and 6(i)). Treatment with NE-100, followed by treatment with the ER stress inhibitor 4-phenylbutyric acid (4-PBA), also decreased the protein expressions of POSTN and CTGF when compared with NE-100 administration only (Figures 6(h) and 6(j)). Notably, TGF-*β*1 administration increased the expression levels of p-PERK, p-IRE1*α*, and ATF4, respectively (Figures 6(a)–6(d)), but the expression of c-ATF6 was not significantly altered. IRE1*α* appeared to be a downstream mediator of Sig1R action in the activation of cardiac fibroblasts, as cells treated with TGF-*β*1 and NE-100 in the presence of the IRE1*α*-specific inhibitor 4*μ*8C showed reduced expression of POSTN and CTGF when compared with the cells without 4*μ*8C treatment (Figures 6(k) and 6(l)). These findings supported a role for ER stress, and especially the IRE1*α* signaling, in the decreased expression of Sig1R in activated cardiac fibroblasts.

### 3.5. Stimulation of Sig1R Ameliorates the Autophagic Flux Impairment in Activated Cardiac Fibroblasts

**Figure 7:**
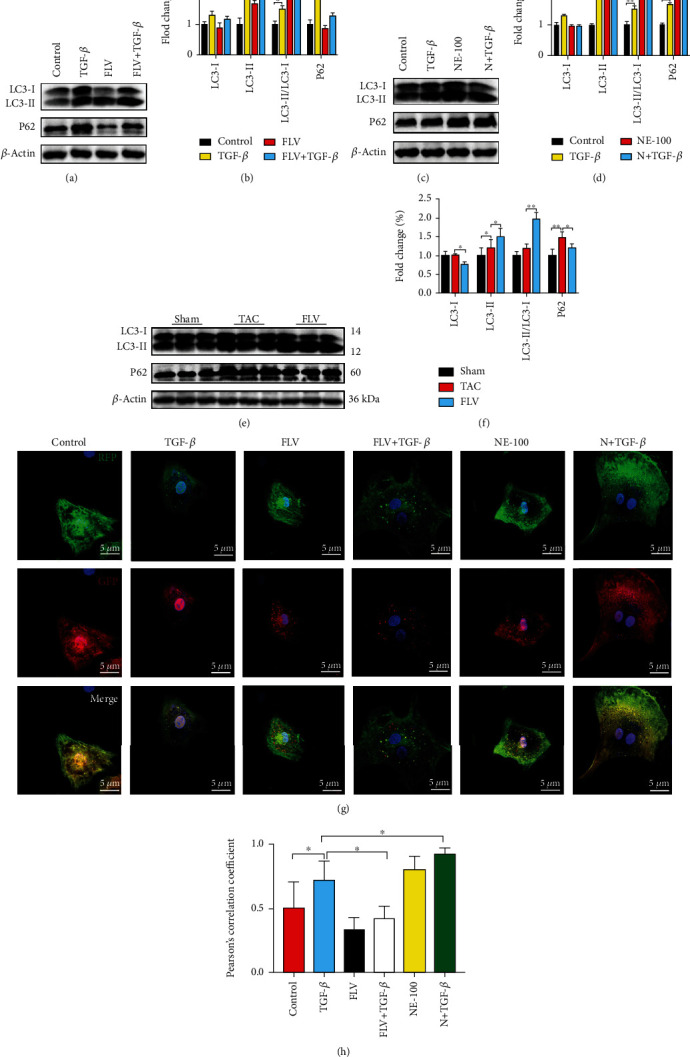
Sig1R protects against cardiac fibrosis by attenuating autophagic flux impairment. (a, b) The representative western blot results of LC3 and p62 in cardiac fibroblasts from control, TGF-*β*1 treatment (TGF-*β*) groups, fluvoxamine treatment (FLV), or fluvoxamine combined with TGF-*β*1 treatment (FLV+TGF-*β*) groups. *n* = 3; (c, d) The representative western blot results of LC3 and p62 in cardiac fibroblasts from control, TGF-*β*1 treatment (TGF-*β*) groups, NE-100 treatment (NE-100), or NE-100 combined with TGF-*β*1 treatment (N+TGF-*β*) groups. *n* = 3; (e, f) The representative western blot results of LC3 and p62 in mice heart tissue from sham-operated (Sham), TAC, and intraperitoneal injection with fluvoxamine after TAC (FLV) groups. *n* = 6; (g, h) The mRFP-GFP-LC3 expressing cells were visualized by confocal microscopy. Merged fluorescence from RFP and GFP was assessed with Pearson's correlation coefficient, and 20 cells were used for quantification in each group. Scale bar = 5 *μ*m. Shown are representative pictures, *p* value was determined by one-way ANOVA with Tukey post hoc analysis. ^∗^*p* < 0.05, ^∗∗^*p* < 0.01, ^∗∗∗^*p* < 0.001. Data represent the mean ± SEM.

**Figure 8:**
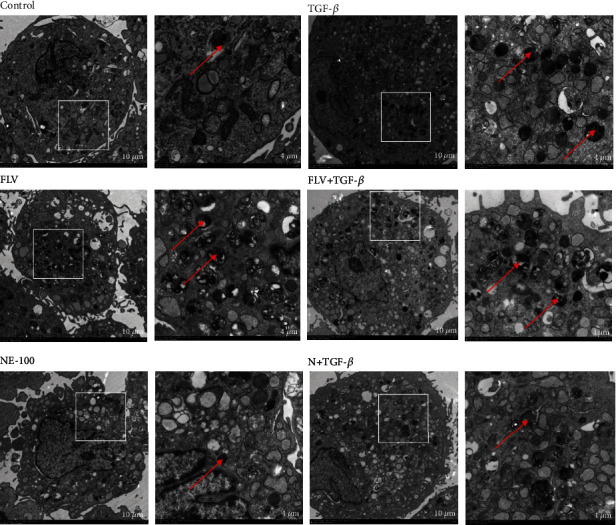
Sig1R protects against cardiac fibrosis by regulating autophagic. A representative image autophagosome was observed by transmission electron microscope in cardiac fibroblasts. Scale bar: 10 *μ*m and 4 *μ*m.

Sig1R modulates some critical steps in the process of autophagy, and measurement of the LC3-II/LC3-I ratio and P62 expression confirmed that autophagic flux was impaired in activated cardiac fibroblasts and fibrosis heart tissue (Figures 7(a)–7(f)). However, the LC3-II/LC3-I ratio was higher in the FLV+TGF-*β* group than in the TGF-*β* group, but P62 was decreased in the FLV+TGF-*β* group (Figures 7(a) and 7(b)). Conversely, NE-100 aggravated the autophagic influx impairment induced by TGF-*β*1 treatment (Figures 7(c) and 7(d)). Additionally, *in vivo* study, the stimulation of fluvoxamine also attenuated the autophagic flux impairment (Figures 7(e) and 7(f)). As shown in Figures 7(g) and 7(h), activated cardiac fibroblasts transfected with mRFP-GFP-LC3 adenovirus showed more autophagosomes (yellow dots) and fewer autolysosomes (red dots). Notably, the numbers of autophagosomes were reduced, and autolysosomes were increased in fluvoxamine pretreated GFP-mRFP-LC3-transfected cardiac fibroblasts, indicating that fluvoxamine restored the autophagic flux in activated cardiac fibroblasts. To further investigate the impact of Sig1R on autophagy, we employed TEM to examine the presence of autophagosomes in cardiac fibroblasts. Relative to the TGF-*β* group, this analysis revealed a significant increase in the number of autophagosomes in the FLV+TGF-*β* group, and the number of autophagosomes is significantly reduced in the N+TGF-*β* group (Figure 8).

## 4. Discussion

**Figure 9:**
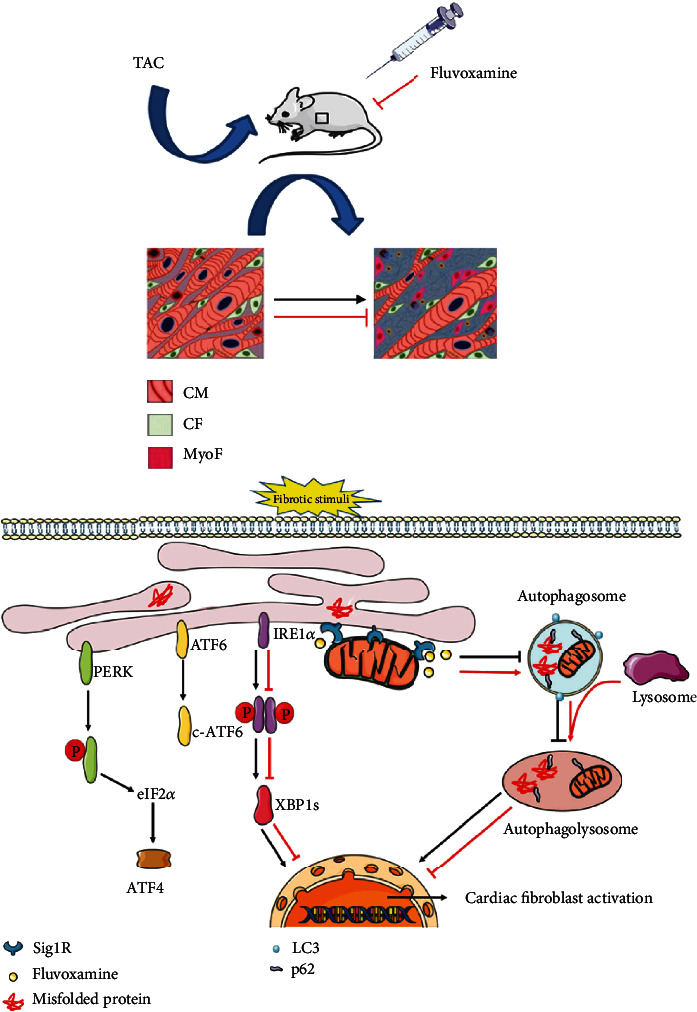
Schematic representation of Sig1R protecting against cardiac fibrosis by regulating IRE1 pathway and autophagic flux. In pressure-overload-induced cardiac fibrosis or TGF-*β*1-induced cardiac fibroblast activation triggers the ER stress and autophagy impairment. Stimulation of Sig1R with fluvoxamine in TAC mice or activated cardiac fibroblasts primed with TGF-*β*1 reduces fibrotic extracellular matrix (ECM) gene expression and cardiac fibrosis.

The present investigation of the role of Sig1R in the activation of cardiac fibroblasts revealed the following major findings illustrated in Figure 9: (1) The expression of Sig1R is decreased in mice heart tissue following TAC operation and in the activation of cardiac fibroblasts induced by TGF-*β*1; (2) Stimulation of Sig1R attenuates the activation of cardiac fibroblasts and cardiac fibrosis; (3) The IRE1*α* pathway mediates the role of Sig1R in the activation of cardiac fibroblasts; (4) Stimulation of Sig1R alleviates the autophagic flux impairment in the activation of cardiac fibroblasts.

Sig1R acts as a pluripotent modulator in many diseases, including Alzheimer's disease and cardiac hypertrophy induced by pressure overload [36, 37], suggesting a pivotal role of Sig1R dysfunction in these diseases. Our previous study showed a decreased expression of Sig1R in hypertrophic rat hearts after TAC [9]. In the present study, a similar reduction in Sig1R expression was observed in mouse hearts showing TAC-induced cardiac fibrosis and in activated cardiac fibroblasts. To determine the role of Sig1R in the activation of cardiac fibroblast, we tried to use fluvoxamine, an agonist of Sig1R *in vivo* and *in vitro*, to observe its effect on the activation of cardiac fibroblast. The results showed that fluvoxamine increased the level of Sig1R in cardiac fibroblasts. Upregulation of Sig1R activity can not only significantly improve the cardiac function decline and cardiac fibrosis *in vivo* but also inhibit the proliferation and migration ability of activated cardiac fibroblasts *in vitro*, indicating a potential protective role of Sig1R stimulation against cardiac fibrosis. To further verify this conclusion, we used Sig1R antagonist NE-100 to intervene Sig1R. The results showed that downregulating the activity of Sig1R greatly increased the expression of TGF-*β*1-induced cardiac fibroblast activation and exacerbated the proliferation and migration ability of activated cardiac fibroblasts.

Furthermore, in most diseases, including depression and mental disorders, the level of Sig1R is downregulated, while upregulating its expression can slow the progression of many diseases [38]. Studies have shown that depression can increase the risk of heart failure, as well as morbidity and mortality [39] In turn, cardiovascular disease will also cause severe depression [40]. Because Sig1R is a common target, some scholars have already proposed the combined use of serotonin reuptake inhibitors (SSRI) in the treatment of cardiovascular diseases to reduce its morbidity and mortality [41]. However, there is no report about the role of SSRI in the process of cardiac fibrosis and cardiac fibroblast activation. Fluvoxamine, one of the SSRIs, is a specific agonist of Sig1R and one of the most commonly used drugs in the clinical treatment of depression. In the present study, fluvoxamine attenuates the pressure-overload-induced cardiac fibrosis in mice. Therefore, we recommend using fluvoxamine clinically to treat patients with both cardiovascular disease and depression; of course, this needs more research.

Small molecule drugs are generally considered to have several off-target effects. So, in this study, after blocking the activity of Sig1R with small molecule inhibitors, we further used specific siRNA to silence the Sig1R gene expression. The results showed that silencing Sig1R gene expression aggravated the activation of cardiac fibroblasts, and the results were consistent with the effects of small molecule inhibitors. Although there may be some off-target effects of small molecule drugs, fluvoxamine/NE-100 has been widely used by other scholars in the research work of Sig1R due to its effectiveness in stimulation or blockage of Sig1R [27, 28]. In addition, compared with overexpressing virus vectors such as adenovirus, fluvoxamine has advantages in clinical translations. Therefore, in the subsequent studies, we continued to use small molecule drugs targeting Sig1R as an intervention.

Sig1R is also expressed in lung fibroblasts and hepatic stellate cells [42, 43], but no role has yet been established in lung or hepatic fibrosis. A recent study reveals that the inhibition of Sig1R promotes atrial electrical remodeling, cardiac autonomic remodeling, and atrial fibrosis, and these changes could be attenuated by fluvoxamine [44]. Therefore, further investigations of the function of Sig1R in other types of tissue fibrosis would be worthwhile.

Another major finding of our present study is that the IRE1*α* pathway, one of the three arms of the ER stress pathways, contributes to the Sig1R-mediated activation of cardiac fibroblasts. We found that three pathways downstream of ER stress in heart tissue of cardiac fibrosis induced by pressure overload: IRE1*α*/XBP1, PERK/ATF4, and ATF6 were all activated. While among activated cardiac fibroblasts induced by TGF-*β*1, only the IRE1*α*/XBP1 and PERK/ATF4 pathways were activated, with no significant changes observed in the ATF6 pathway. We analyzed and ascribed this phenomenon to different expressions of various types of cells contained in mouse heart tissues, including expressions of cardiomyocytes, cardiac fibroblasts, and vascular endothelial cells. IRE1*α* can splice the mRNA of transcription factor X-box binding protein 1 (Xbp1), which produces the functionally active spliced form of Xbp1 (Xbp1s). Xbp1s, in turn, translocate into the nucleus to induce the expression of other ER chaperones and antioxidant proteins [44]. IRE1*α* resides mainly in the MAM [45]. At the MAM, Sig1R binds with and interacts with IRE1 [18]. A recent study has reported that Sig1R restricts the endonuclease activity of IRE1 against inflammation [35], which agrees with our finding that the IRE1 pathway mediates the stimulatory effect of Sig1R on cardiac fibroblast activation.

Most importantly, due to its location in the MAM and ER membranes, Sig1R exhibits a critical role in autophagy [46]. Owing to the highly dynamic process involved in autophagosome synthesis, cargo recognition and transport, autophagosome-lysosome fusion, and cargo degradation, the quantifying of autophagy becomes a challenge. It is critical to consider not only the number of autophagosomes within the cell but also the autophagic degradative activity, autophagic flux [47, 48]. Therefore, autophagic flux is a commonly used index to monitor the process of autophagy. A previous study reports that Sig1R ablation impairs autophagosome clearance [49]. In the present study, our results have confirmed and extended their results. Sig1R stimulation attenuates the autophagic flux impairment in activated cardiac fibroblasts, whereas Sig1R inhibition aggravates the impairment.

Autophagy plays an important role in cardiac fibrosis, as well as in other fibrotic diseases [50]. Zhang et al. [51] reported that the tribbles pseudokinase 3 (TRIB3) mediates autophagy impairment by not only suppressing autophagic degradation but also promoting the activation of hepatic stellate cells (HSCs). Notably, restoration of the autophagic flux in hepatocytes and HSCs has potent protective effects against hepatic fibrosis [51]. Another study has shown that the activation of Sig1R increases nuclear factor erythroid-2-related factor 2 antioxidative response element (Nrf2-ARE) binding activity in retinal cone photoreceptor cells, and Sig1R participates in protecting cells from electrophilic or oxidative stress by regulating the expression of antioxidant genes, suggesting an involvement of Sig1R in Nrf2 signaling [52]. Sig1R also reduces the production of reactive oxygen species (ROS) by enhancing the signaling of Nrf2 [53]. Constitutive activation of Nrf2 augments autophagosome formation and promotes autophagic flux in the heart after TAC [54]. Therefore, we speculate that the restoration of autophagic flux in cardiac fibroblasts by Sig1R agonists may also be mediated by the Nrf2 signaling pathway.

Some studies have identified the critical roles of Sig1R in mediating cell survival by a regulation of the interplay between apoptosis and autophagy [55]. The interaction between ER stress (and especially the IRE1 pathway) and autophagy in the activation of cardiac fibroblast clearly needs further study.

Taken together, the findings presented here indicate that the stimulation of Sig1R attenuates the activation of cardiac fibroblasts and cardiac fibrosis induced by pressure overload by alleviating the IRE1 pathway and autophagy impairment. Overall, these results suggest that Sig1R might be a promising therapeutic target for cardiac fibrosis treatments.

## Figures and Tables

**Table 1:** The reaction system of reverse transcript.

Reagent	Volume (*μ*L)
5 × RT buffer	4.0
Primer mixture	1.0
ReverTra Ace	1.0
1.5 *μ*g RNA	X
RNAase-free ddH2O	(14-X)
Total volume:	20 *μ*L

**Table 2:** Rat primer sequences and amplicon sizes for RT-PCR.

Genes	Primer sequence (5′-3′)
COL-1	F: 5′ACGTCCTGGTGAAGTTGGTC3′ R: 5′TCCAGCAATACCCTGAGGTC3′

CTGF	F: 5′CAGGGAGTAAGGGACACGA3′ R: 5′ACAGCAGTTAGGAACCCAGAT3′

*β*-Actin	F: 5′CCUCUCCUUUGGACUGUAU3′ R: 5′ATGCCACAGGATTCCATACCC3′

TGF-*β*	F: 5′TGAGTGGCTGTCTTTTGACG3′ R: 5′ACTGAAGCGAAAGCCCTGTA3′

Sig1R	F: 5′ATTTCTCTACTCGCTGGGACTC3′ R: 5′GAGCTGTGTCTGGATGTATGTG3′

**Table 3:** Mouse primer sequences and amplicon sizes for RT-PCR.

Genes	Primer sequence (5′-3′)
POSTN	F: 5′TGGTATCAAGGTGCTATCTGCG3′ R: 5′AATGCCCAGCGTGCCATAA3′

CTGF	F: 5′GGACACCTAAAATCGCCAAGC3′ R: 5′ACTTAGCCCTGTATGTCTTCACA3′

COL-1	F: 5′TAAGGGTCCCCAATGGTGAGA3′ R: 5′GGGTCCCTCGACTCCTACAT3′

TGF-*β*	F: 5′CTTCAATACGTCAGACATTCGGG3′ R: 5′GTAACGCCAGGAATTGTTGCTA3′

Sig1R	F: 5′GGCACCACGAAAAGTGAGGT3′ R: 5′AGAACAGGGTAGACGGAATAACA3′

*β*-Actin	F: 5′GTGACGTTGACATCCGTAAAGA3′ R: 5′GCCGGACTCATCGTACTCC3′

## Data Availability

All data included in this study are available upon request by contact with the corresponding author.
